# Hypoxia stimulates the migration and invasion of osteosarcoma via up-regulating the NUSAP1 expression

**DOI:** 10.1515/med-2020-0180

**Published:** 2021-07-22

**Authors:** Ling Zhang, Jingtao Song, Xu Xin, Donghong Sun, Huiting Huang, Yang Chen, Tao Zhang, Yiming Zhang

**Affiliations:** Department of Orthopedics, Huabei Petroleum General Hospital, Huizhan Road, Renqiu 062552, Hebei, China; Department of Orthopedics, Tianjin Beichen District Chinese Medicine Hospital, Tianjin 300400, China; Department of Clinical Medicine, Tianjin Medical University, Tianjin 300070, China

**Keywords:** osteosarcoma, hypoxia, HIF-1α, NUSAP1, migration, invasion

## Abstract

Osteosarcoma is a highly aggressive malignant tumor, which most commonly occurs in children and adolescents. This study aims to reveal that hypoxia promotes the invasion of osteosarcoma cells by up-regulating the expression of NUSAP1. The expression of HIF-1α and NUSAP1 was significantly up-regulated in MG63 cells cultured in hypoxia for 6–36 h. Furthermore, hypoxia induced the migration and invasion of MG63 cells and regulated the level of E-cad, N-cad, Vimentin, Snail, Slug, MMP2, and MMP9 proteins. Importantly, knockdown of NUSAP1 inhibited hypoxia-induced cell migration and invasion. In the hypoxia microenvironment, the addition of HIF-1α inhibitor or the transfection of siRNA specifically targeting HIF-1α significantly reduced the expression of HIF-1α and NUSAP1 and markedly inhibited the migration and invasion of MG63 cells under the hypoxia microenvironment. In conclusion, hypoxia induced the expression of NUSAP1 in a HIF-1α-dependent manner, stimulating the migration and invasion of MG63 cells.

## 1 Introduction

Osteosarcoma is a highly aggressive malignant tumor, which most commonly occurs in children and adolescents [1]. The combination of neoadjuvant chemotherapy and traditional surgical resection is the main treatment strategy for osteosarcoma, which improves the overall survival of patients with local osteosarcoma [2]. However, osteosarcoma has a high recurrence rate and is prone to lung metastasis. For metastatic and recurrent osteosarcoma, the combination of surgery and chemotherapy cannot produce satisfactory outcomes [3]. In other words, the clinical outcome of patients with osteosarcoma has not improved significantly. This stagnation of therapeutic advances may be attributed to the unclear mechanism of the osteosarcoma occurrence and metastasis [4]. Therefore, understanding the specific mechanisms of biomolecules in the occurrence and metastasis of osteosarcoma is important to improve the prognosis of patients with osteosarcoma.

Intratumoral hypoxia is a typical feature of solid tumors [5]. This is mainly due to the increase in oxygen consumption caused by the rapid growth of tumor mass and the limited blood supply caused by newly formed vascular malformations. Hypoxia is a vital component of the tumor microenvironment, which is closely related to cell proliferation, tumor invasion, angiogenesis, and distant metastasis [6,7]. The adaptation of tumor cells to hypoxia led to the selection of tumor heterogenous and resistant clones, which evolved into more aggressive phenotypes and resistance to multiple therapeutic drugs [8]. Tumor metastasis and drug resistance caused by hypoxia have also been demonstrated in osteosarcoma [9,10]. However, further understanding of hypoxia-driven metastasis mechanisms is needed.

Nucleolar and spindle-associated protein 1 (NUSAP1) can control the cell cycle progression by promoting the accumulation of microtubules [11,12,13]. The high expression of NUSAP1 has been found in a variety of tumor types and is closely related to tumor cell proliferation, apoptosis, and drug resistance [14,15,16]. However, the role of NUSAP1 in the hypoxia response of osteosarcoma has not been reported. Although, one study has found that hypoxic stress stimulates the rapid translation of NUSAP1 in pancreatic cancer cells [17].

In this study, we found that hypoxia induced the expression of NUSAP1, thereby stimulating the migration and invasion of MG63 cells. Additionally, hypoxia-induced NUSAP1 expression and MG63 cell migration and invasion are HIF-1α dependent.

## 2 Materials and methods

### 2.1 Cell lines and cell culture

The human osteosarcoma cell line MG63 was purchased from the Institute of Biochemistry and Cell Biology, Chinese Academy of Sciences (Shanghai, China). Cells were maintained in Dulbecco’s Modified Eagle’s Medium (DMEM) (Gibco, Grand Island, NY) supplemented with 10% fetal bovine serum (FBS) (Thermo Fisher Scientific, Waltham, MA), 100 U/mL penicillin, and 100 mg/mL streptomycin. For the normoxic culture, cells were incubated at 37 °C with 5% CO_2_, 20% O_2_, and 75% N_2_ in a humidified incubator (Thermo Fisher Scientific, Waltham, MA). For the hypoxia culture, cells were exposed at 37°C with 1% O_2_, 5% CO_2_, and 94% N_2_ in a humidified incubator.

### 2.2 Cell transfection and treatment

MG63 cells were transfected with siRNA targeting NUSAP1 or negative control siRNA (Ruibo, Guangzhou, China) using Lipofectamine 2000 reagent (Invitrogen, Carlsbad, CA) following the manufacturer’s instructions. HIF-1α inhibitor (LW6) was obtained from MedChemExpress (Cat. No. HY-13671).

### 2.3 Transwell assay

The migration and invasion abilities of MG63 cells were detected by the Transwell assay. For cell migration, 2,000 cells maintained in 200 μL of serum-free medium were seeded in the upper well of the Transwell chamber (8 μm pore size; Corning, Shanghai, China). About 600 μL of DMEM containing 10% FBS was loaded into the lower well. After 24 h of incubation, cells that did not migrate through the pores were carefully wiped with a cotton swab. The cells located on the lower surface of the chamber were fixed with 4% paraformaldehyde for 30 min and stained with 0.1% crystal violet for 30 min at room temperature. The stained cells were counted under a light microscope (Olympus, Tokyo, Japan) from five random fields. For cell invasion, the upper chamber was coated with the Matrigel (BD Biosciences, San Jose, CA). Then, the other operations were consistent with cell migration experiments.

### 2.4 Western blotting

Proteins were lysed from MG63 cells with RIPA buffer containing the protease and phosphatase inhibitors and quantified using the BCA assay (Beyotime, Shanghai, China). An equal amount of protein from each sample was loaded on a 10% SDS-PAGE gel and electrophoresed. Then, the proteins were transferred to a PVDF membrane (EMD Millipore, Billerica, MA), following block with 5% fat-free milk for 1 h at room temperature. The blots were incubated with primary antibodies overnight at 4°C. After washing three times with TBST, the membrane was incubated with HRP-conjugated secondary antibodies, and the immunoblots were visualized using ECL detection kit (Thermo Fisher Scientific). Software QUANTITY ONE was used to measure the intensity of bands. β-actin was used as the reference.

The primary antibodies used in this study were as follow: anti-hypoxia-inducible factor 1α (HIF-1α) (ab1, mouse monoclonal), anti-NUSAP1 (ab169083, mouse polyclonal), anti-N-cadherin (ab18203, rabbit polyclonal), anti-Vimentin (ab8978, mouse monoclonal), anti-Snail (ab229701, rabbit monoclonal), anti-Slug (ab51772, mouse monoclonal), anti-MMP2 (ab92536, rabbit monoclonal), anti-MMP9 (ab38898, rabbit polyclonal), and anti-β-actin (ab8226, mouse monoclonal) were purchased from Abcam. Anti-E-cadherin (20874-1-AP, rabbit polyclonal) was purchased from Proteintech.

### 2.5 Statistical analysis

Statistical analysis was performed with GraphPad Prism 5 (GraphPad Software, La Jolla, CA). All data were expressed as means ± SEM from three or more independent experiments. The differences between the groups were determined by Student’s *t*-test or one-way ANOVA and considered significant at *P* < 0.05.

## 3 Results

### 3.1 Hypoxia induces NUSAP1 expression in MG63 cells

**Figure 1:**
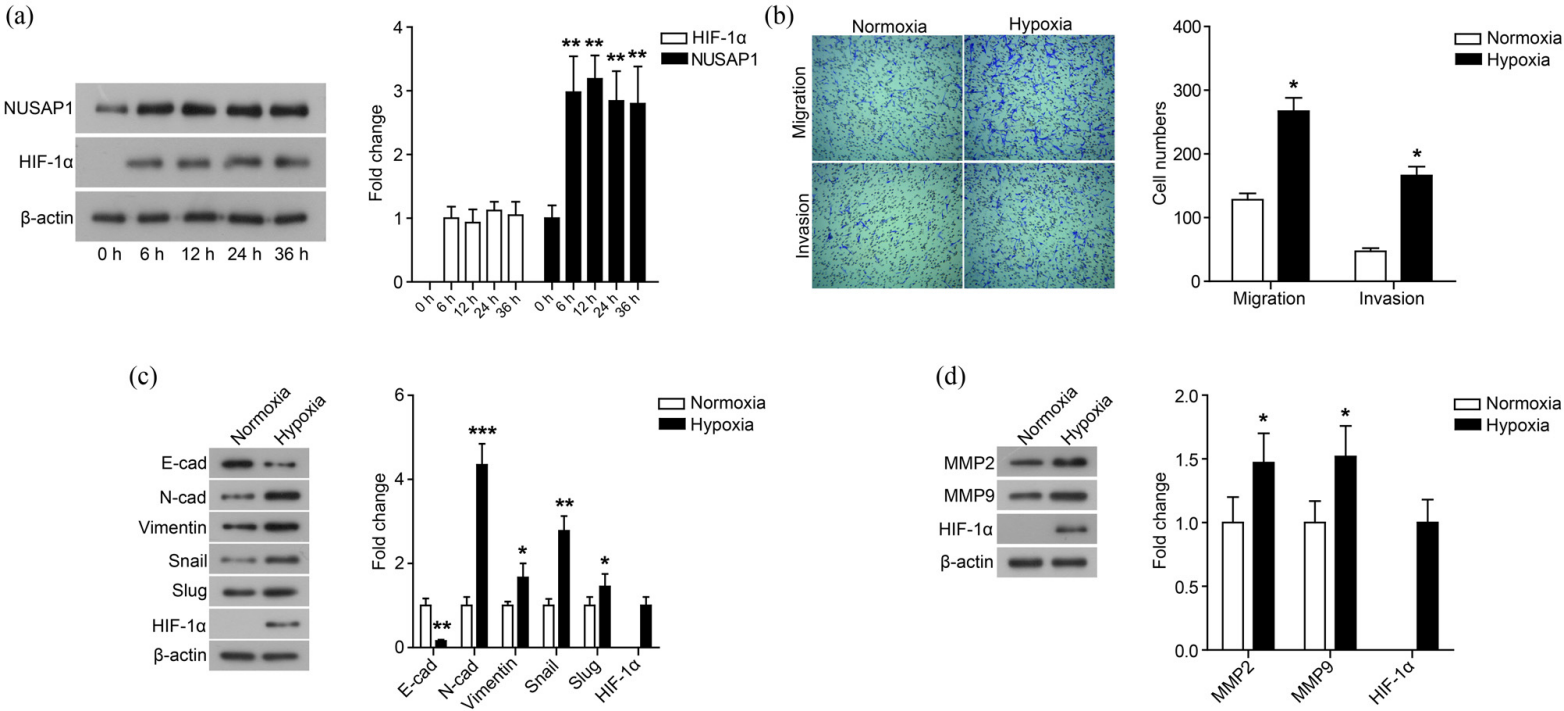
Hypoxia induces NUSAP1 expression and stimulates the migration and invasion of MG63 cells. (a) The expression of HIF-1α and NUSAP1 in MG63 cells cultured with hypoxia for 0, 6, 12, 24, and 36 h was detected using western blot. (b) The migration and invasion of MG63 cells cultured with normoxia or hypoxia for 24 h was measured using transwell assay. (c) The expression of EMT-related proteins in MG63 cells cultured with normoxia or hypoxia for 48 h was detected using western blot. (d) The expression of MMP family membranes in MG63 cells cultured with normoxia or hypoxia for 48 h was detected using western blot. All experiments were independently carried out in three replicates. *P < 0.05, **P < 0.01, ***P < 0.001, compared with normoxia group.

We investigated the effects of hypoxia on the expression of NUSAP1 in human osteosarcoma cell line MG63. As shown in Figure 1a, the expression of NUSAP1 was significantly up-regulated in MG63 cells cultured in hypoxia for 6–36 h. In addition, the level of HIF-1α increased significantly under the hypoxia microenvironment for 6–36 h.

### 3.2 Hypoxia stimulates MG63 cell migration and invasion

Subsequently, the effect of hypoxia on the migration and invasion of MG63 cells was measured by the Transwell assay. As shown in Figure 1b, the number of migrating and invading cells increased after exposure to hypoxia (*P* < 0.05). Furthermore, the hypoxia microenvironment regulated the expression of EMT-related proteins. As shown in Figure 1c, the hypoxia microenvironment down-regulated the expression of E-cad of MG63 cells, while up-regulating the level of N-cad, Vimentin, Snail, and Slug (*P* < 0.05). We also found that the expression of MMP2 and MMP9 significantly increased under the hypoxia microenvironment (Figure 1d, *P* < 0.05).

### 3.3 Knockdown of NUSAP1 represses the migration and invasion of MG63 cells

**Figure 2:**
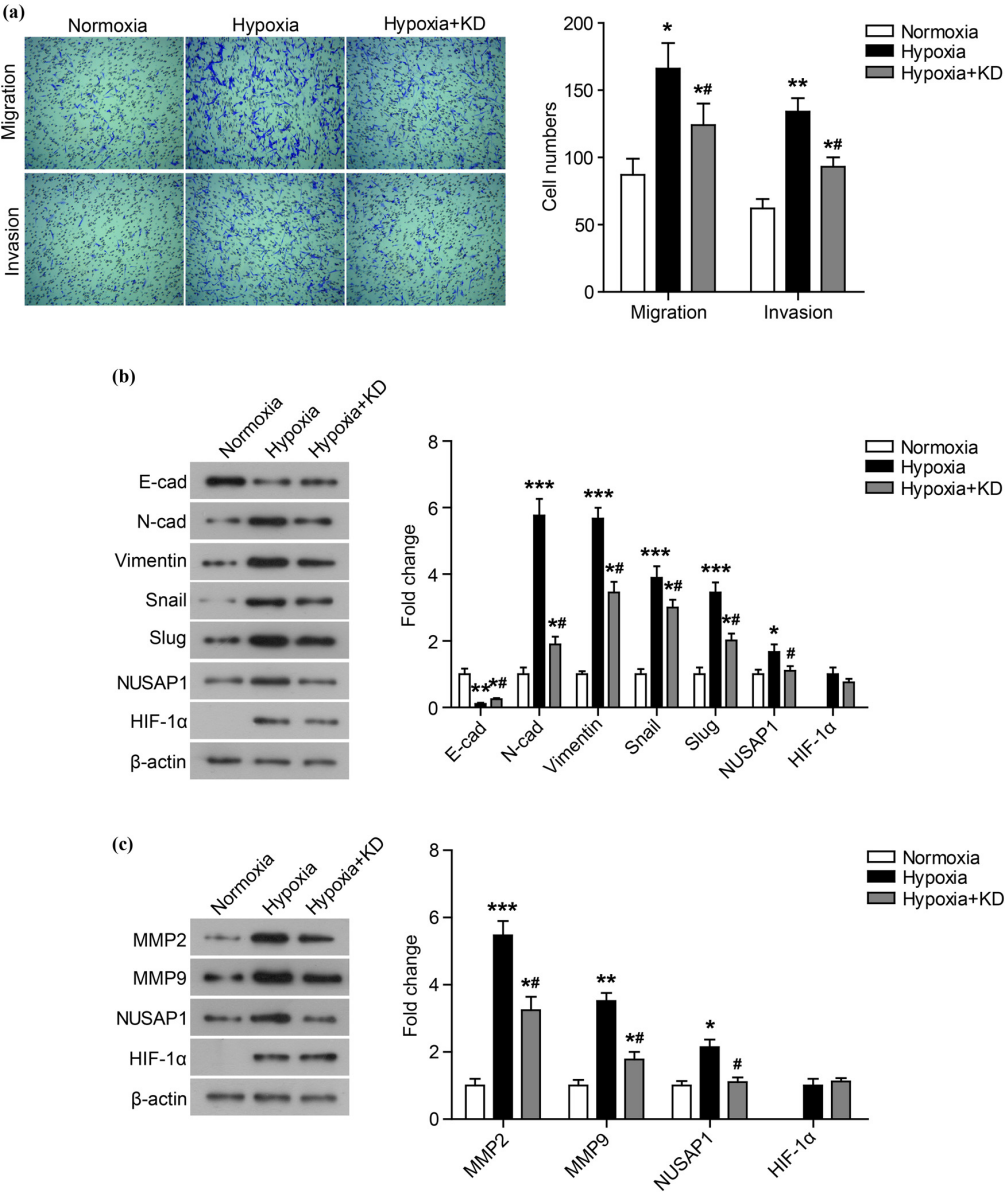
Knockdown of NUSAP1 represses the migration and invasion of MG63 cells under hypoxia. (a) The migration and invasion of MG63 cells whose NUSAP1 expression was knocked down (Hypoxia + KD) under hypoxia condition was measured using transwell assay. (b) The expression of EMT-related proteins was detected using western blot. (c) The expression of MMP family membranes was detected using western blot. All experiments were independently carried out in three replicates. *P < 0.05, **P < 0.01, ***P < 0.001, compared with normoxia group; ^#^ *P* < 0.05, compared with Hypoxia group.

To investigate whether hypoxia-induced cell migration and invasion are related to the hypoxia-induced high expression of NUSAP1, NUSAP1 expression was knocked down by transfection of siRNA-NUSAP1 under hypoxia condition. As shown in Figure 2a, after knocking down the NUSAP1 expression, the number of migrating and invading cells under hypoxia conditions was markedly reduced. Knockdown of NUSAP1 significantly suppressed the expression of N-cad, Vimentin, Snail, Slug, MMP2, and MMP9, while it markedly promoted the expression of E-cad (Figure 2b and c, *P* < 0.05).

**Figure 3:**
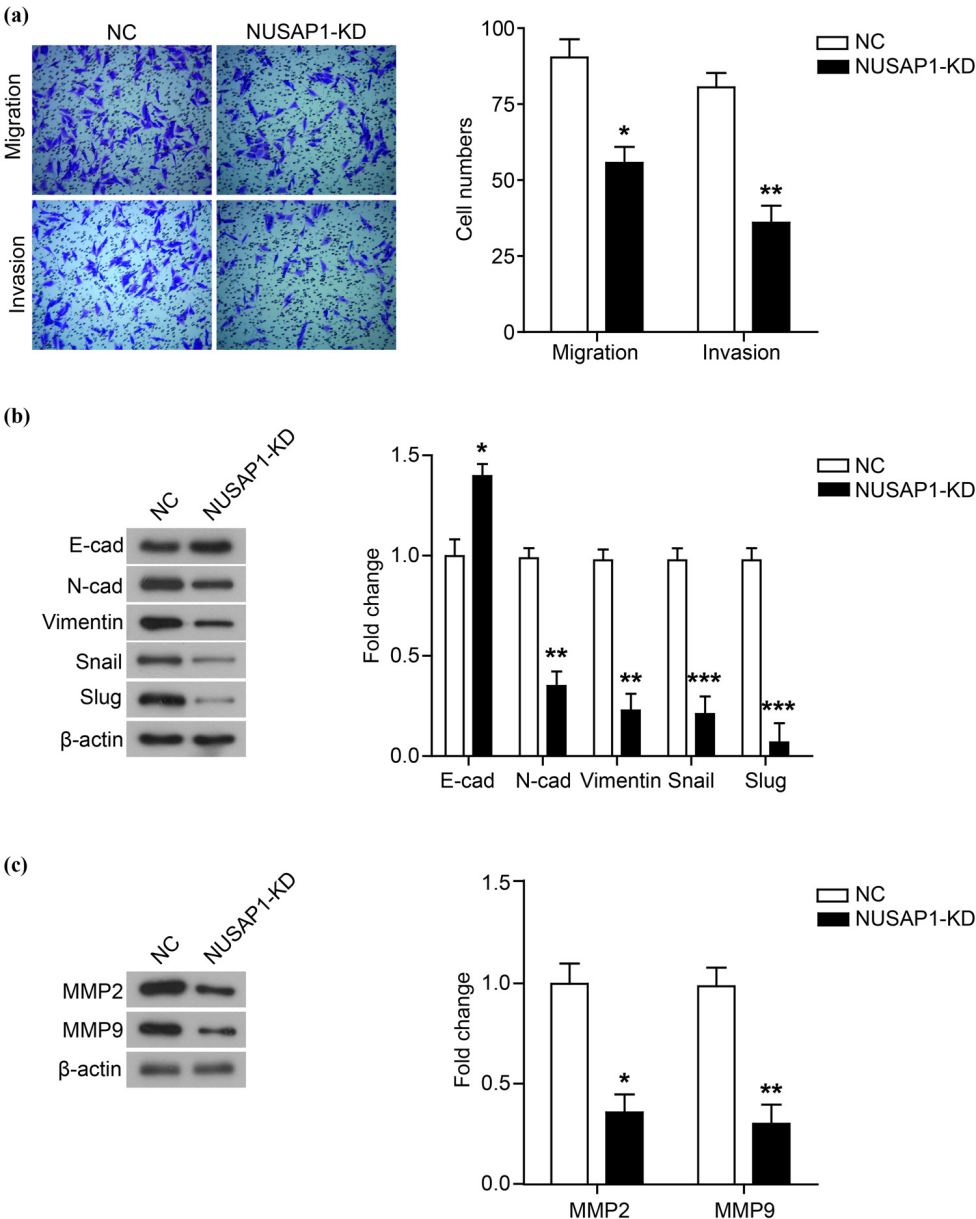
Knockdown of NUSAP1 represses the migration and invasion of MG63 cells. (a) The migration and invasion of MG63 cells whose NUSAP1 expression was knocked down (NUSAP1-KD) under normoxia condition was measured using transwell assay. (b) The expression of EMT-related proteins was detected using western blot. (c) The expression of MMP family membranes was detected using western blot. All experiments were independently carried out in three replicates. *P < 0.05, **P < 0.01, ***P < 0.001, compared with NC group.

In addition, we also measured the effect of down-regulation of NUSAP1 expression on MG63 cell migration and invasion under normoxia. As shown in Figure 3, knockdown of NUSAP1 inhibited cell migration and invasion and regulated the expression of EMT-related proteins and MMP proteins.

### 3.4 Hypoxia increases NUSAP1 expression and MG63 cell migration and invasion is HIF-1α dependent

**Figure 4:**
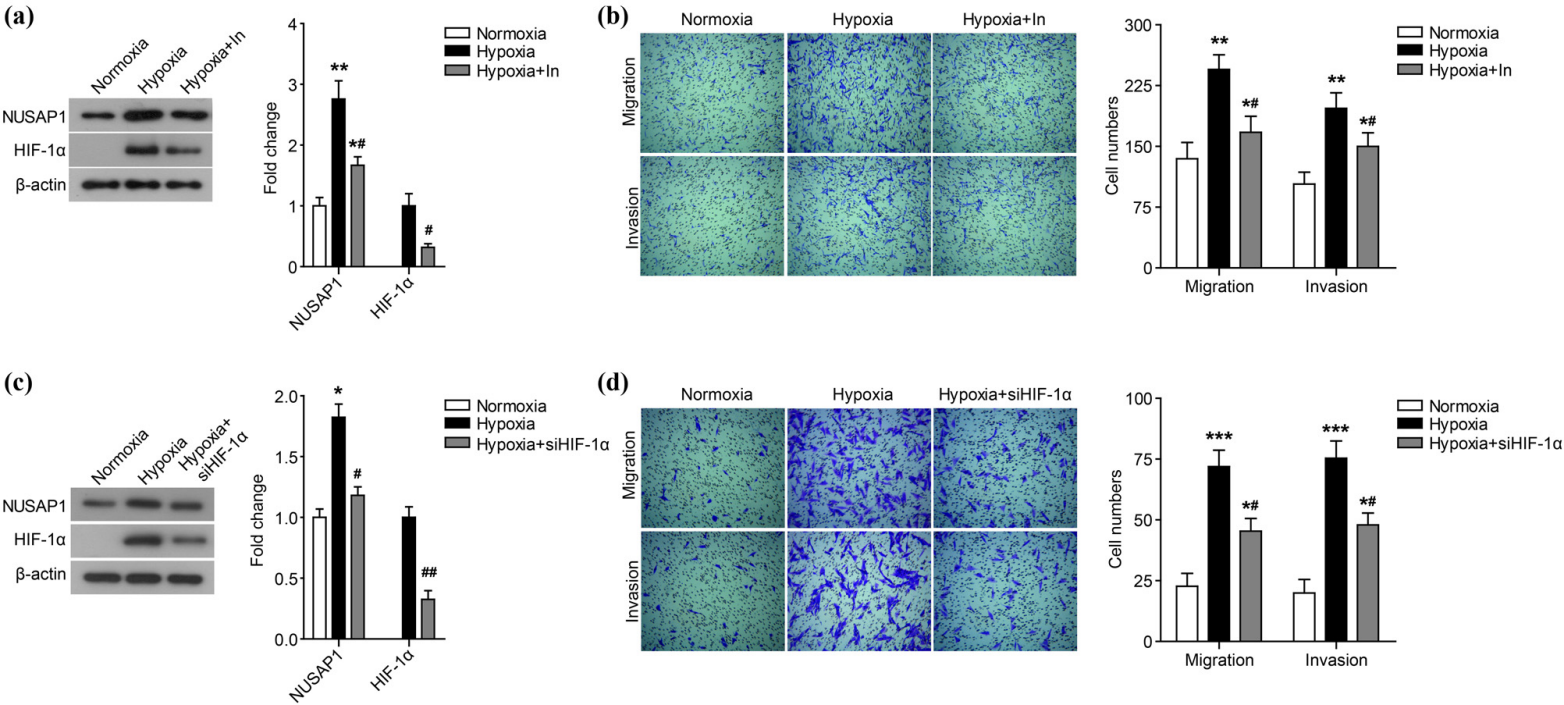
Hypoxia increases NUSAP1 expression and MG63 cell migration and invasion is HIF-1α dependent. The expression of HIF-1α and NUSAP1 in MG63 cells incubated with HIF-1α inhibitor (Hypoxia + In) (a) or siRNA targeting HIF-1α (siHIF-1α) (c) under hypoxia condition was detected using western blot. The migration and invasion of MG63 cells incubated with HIF-1α inhibitor (b) or siRNA targeting HIF-1α (siHIF-1α) (d) under hypoxia condition was measured using transwell assay. All experiments were independently carried out in three replicates. *P < 0.05, **P < 0.01, ***P < 0.001, compared with normoxia group; ^#^ *P* < 0.05, compared with Hypoxia group.

HIF-1α is the central transcription factor that regulates the transcription of hypoxia-responsive genes and the adaptive response of cells to hypoxia. Therefore, we further evaluated whether the hypoxia-induced up-regulation of NUASP1 expression is HIF-1α dependent. In the hypoxia microenvironment, the addition of HIF-1α inhibitor (Figure 4a) and the transfection of siRNA specifically targeting HIF-1α (siHIF-1α) (Figure 4c) can significantly reduce the expression of HIF-1α and NUSAP1 (*P* < 0.05). Additionally, the addition of HIF-1α inhibitor and the transfection of siHIF-1α both markedly inhibited the cell migration and invasion under the hypoxia microenvironment (Figure 4b and d, *P* < 0.05).

## 4 Discussion

Osteosarcoma is a common primary malignant tumor, accounting for more than 10% of solid cancers in children and adolescents [18]. Although the treatment of osteosarcoma has made great progress in the past 20 years, the overall survival rate of osteosarcoma patients has not improved due to metastasis and recurrence [19]. Therefore, it is necessary to understand its underlying biological reasons to improve the outcome of osteosarcoma treatment. In this study, we found that hypoxia can promote the migration and invasion of osteosarcoma cells by up-regulating the expression of NUSAP1. These findings may provide a valuable target for the treatment of osteosarcoma.

Hypoxia is an important prognostic and driven factor for many types of tumors [20,21]. In this study, we found that hypoxia induced the expression of NUSAP1. Furthermore, one study has shown that hypoxia stimulates the rapid translation of NUSAP1 in pancreatic cancer cells [17]. Additionally, we also found that hypoxia promoted the migration and invasion of osteosarcoma cells and regulated the expression of EMT- and MMP-related proteins. Hypoxia-induced cell migration and invasion were related to the high expression of NUSAP1 induced by hypoxia. After knocking down the expression of NUSAP1, the number of migrating and invading cells under hypoxia was markedly reduced.

The members of the HIF family are oxygen sensors that mediate the response of mammalian cells to hypoxia [22]. The members of this family contain an oxygen-sensitive HIF-α subunit and a constitutively expressed HIF-β subunit [22]. HIF-1α is widely used as a marker of poor prognosis in cancer patients. It can act as a signaling center, transcriptionally regulating the expression of many transcription factors and signaling molecules that play a key role in tumorigenesis [22]. Additionally, HIF-1α plays an important role in the response of cells to hypoxia by inducing glycolysis and angiogenesis [23]. Some studies have also shown that HIF-1a is up-regulated in osteosarcoma and is associated with the metastasis and poor prognosis [5,24,25]. In this study, we found that the increased NUSAP1 expression and MG63 cell migration and invasion induced by hypoxia were HIF-1α dependent. The results of the western blot showed that the addition of HIF-1α inhibitor or the transfection of siRNA targeting HIF-1α significantly reduced the expression of HIF-1α and NUSAP1 in the hypoxia microenvironment and markedly inhibited the cell migration and invasion. We speculated that NUSAP1 was the downstream target of HIF-1α transcriptional regulation and participates in the regulation of osteosarcoma cell migration and invasion in a HIF-1α-dependent manner.

We reported for the first time the role of NUSAP1 in osteosarcoma, which is consistent with its role in other types of tumors. NUSAP1 plays an oncogene role in tumors, which is mainly involved in regulating tumor cell proliferation and apoptosis [14,15,16]. This study also demonstrates the link between NUSAP1 and tumor cell migration and invasion.

In conclusion, hypoxia induced the NUSAP1 expression in a HIF-1α-dependent manner in MG63 cells, which stimulated the migration and invasion. HIF-1α and NUSAP1 were valuable targets for the treatment of osteosarcoma.
